# Field evaluation of two mosquito traps in Zhejiang Province, China

**DOI:** 10.1038/s41598-020-80618-1

**Published:** 2021-01-11

**Authors:** Juan Hou, Yinping Wu, Zhiyuan Mao, Xinhong Zhu, Yuyan Wu, Qinmei Liu, Jinna Wang, Tianqi Li, Zhenyu Gong, Xuanjun Dong, Zhen Wang

**Affiliations:** 1 Zhejiang Provincial Center for Disease Control and Prevention, 3399 Binsheng Road, Hangzhou City, Zhejiang Province China; 2 Yiwu Center for Disease Control and Prevention, 48 Xuefeng East Road, Yiwu City, Zhejiang Province China; 3 MPH Department, College of Veterinary Medicine, Cornell University, Ithaca, NY, USA

**Keywords:** Health occupations, Medical research

## Abstract

Mosquito-borne Diseases are a common but severe public health threat. However, there is a lack of consensus on the effect of different mosquito trapping devices in China. This study firstly compared the BGM trap with the CDC light trap, commonly used in Chinese mosquito surveillance. Field trials of traps' efficiency were conducted in Yiwu city, China, from May 21st, 2018 to November 31st, 2018. Sixty-five comparisons were completed in five different biotopes (an urban residential area, a rural residential area, a park, a hospital, and a pig shelter). Concerning the number of mosquitoes per trap, the BGM trap outperformed three out of five biotopes. In contrast, the CDC light trap only showed better performance in the pig shelter. For specific species, the BGM trap outperformed in capturing *Ae. albopictus*, while the CDC light trap caught significantly more *Cx. tritaeniorhynchus*. Regarding *Ae. albopictus* and *Cx. pipiens* s.l*.* surveillance, the BGM trap is more suitable. The BGM trap shows significantly higher or similar efficiency than the CDC light trap in trapping common mosquito species in China, except in the pig shelter. Therefore, we recommend that Chinese researchers and public health practitioners use the BGM trap in future mosquito surveillance.

## Introduction

Mosquitoes can transmit infectious pathogens to human through bites, serving as vectors of diverse diseases^1^. In the past three decades, China has identified a series of mosquito-borne disease cases, including Dengue, Chikungunya fever, Yellow fever, Zika virus disease, Japanese encephalitis, and Malaria, etc^2,3^. These diseases occurs in a broad geographical range of China that cases have been reported in the coastal area, such as Guangdong Province, and plateau areas, like Tibet and Yunnan Province of this country^4–6^. Among all identified arbovirus disease, Dengue is the most severe public health threat to this nation, as over 650,000 cases had been reported from 1978 to 2008^7^. To date, China has only vaccines, against Yellow fever and Japanese encephalitis, available; however, has no vaccines available for other mosquito-borne disease (i.e., Dengue). Therefore, given the medical importance of mosquitoes, vector surveillance and control is of critical importance in mosquito-borne disease prevention and control.

CDC light trap has been the primary tool of mosquito surveillance in China for decades. Before Dengue emerges, Japanese encephalitis and Malaria were the two heavy burdens of mosquito-borne diseases in China, the incidences of which were as high as 20.92 per 100,000 population and 3000 per 100,000 population in the 1970s, respectively^7,8^. *Culex* mosquitoes are the vectors of Japanese encephalitis, while *Anopheles* mosquitoes are Malaria’s. The light traps show high efficiency in capturing these two mosquito species^9^. At that time, the CDC light trap can perfectly meet the need to monitor *Culex* mosquitoes and *Anopheles* mosquitoes in the five biotopes.

As Dengue emerges in these years, *Aedes albopictus*, the secondary vectors of Dengue virus, become the main target of mosquito surveillance in China, because the population of the primary vector, *Aedes aegypti,* decreased largely in mainland China in recent years^10^. Nevertheless, an analysis conducted by Chinese Center for Disease Control and Prevention reported that CDC light traps’ efficiency in trapping *Ae. albopictus* were only of 0.25 individual per trap per night, lower than the efficiency of human-baited double net traps of 2.95 per trap per hour^11^. Given that, there is an urgent need to replace the current devices in order to improve the efficiency in capturing *Aedes* mosquitoes^12^.

With the technological development, the choice of mosquito surveillance is diversified. The Biogents company has produced a series of mosquito traps: BG-Sentinel (BGS), BG-GAT, BG-Suna, BG-Mosquitaire CO_2_ (BGM), etc. As the benchmark of *Ae. albopictus* trapping devices also showing high efficiency in multiple mosquito species, the BG-Sentinel trap has been recommended by U.S. CDC for mosquito surveillance use over a decade^13–16^. BGM trap is an adaption of BGS trap, which keeps the same mechanism but is more durable^17^. Therefore, BGM is supposedly suitable for long-term mosquito surveillance. However, there is no adequate evidence in China showing BGM’s effectiveness. Our study aims to examine the performance of the BGM and the CDC light trap on common mosquito species in China and determine whether BGM can replace CDC light traps in routine mosquito surveillance.

### Study site

**Figure 1:**
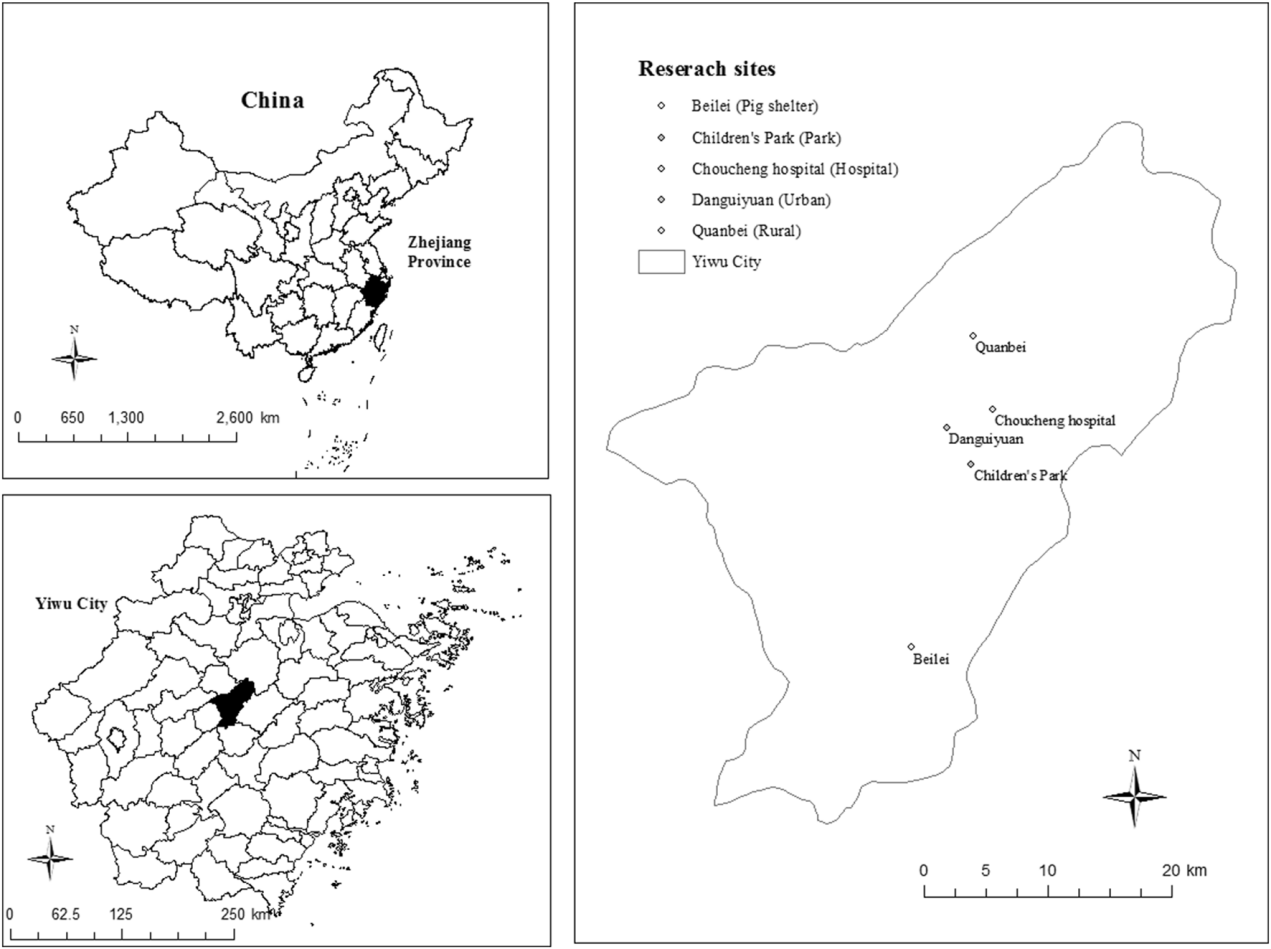
Sampling locations and geographical information about Yiwu City.

Field trials were carried out in Yiwu city, Zhejiang Province, China (119° 49′–120° 17′ E, 29° 02′ 13″ 29° 33′ 40″ N, 56 m above sea level, m.a.s.l.) (Fig. 1). In 2005, the Chinese national disease vector surveillance system (CNDVSS) required that mosquito surveillance tools should be used to monitor five different biotopes—urban residential areas, rural residential areas, hospitals, parks, and animal shelters^18^. Based on the guidance, we choose five different locations within Yiwu city to represent five different kinds of biotopes. Beilei (120° 03′ E, 29° 16′ 55″ N) is a pig farm and hence represents the animal shelter; Quanbei (120° 08′ E, 29° 39′ 16″ N) where is a village represents the rural residential area; Danguiyuan (120° 06′ E, 29° 32′ 48″ N) is an urban neighborhood in Yiwu city’s downtown and therefore represents the urban residential area; Children’s park (120° 07′ E, 29° 29′ 83″ N) is selected as the biotope of park and Choucheng Hospital (120° 09′ E, 29° 33′ 81″ N) is selected as the biotope of hospital (The rural residential zone and the urban residential zone are abbreviated as Urban and Rural, respectively, in this article's charts.). The least distance between any pair of biotopes is 3.44 km (Table 1). Yiwu city has a typical hilly landform located in the subtropical monsoon climate zone, with an average annual temperature of 17 °C and annual precipitation of 1100–1600 mm^19^. The local climate is ideal for mosquito breeding, and adult mosquitoes are active from April to November each year^20^. Known as the largest wholesale market globally, Yiwu city has approximately 500,000 foreign businessmen come for trade annually, and over 15,000 foreigners hold permanent residency^19^. Population influx increases the population density of Yiwu city, which indirectly facilitating pathogens’ transmission and increasing the risk of mosquito-borne disease outbreaks^21^.

**Table 1:** Distance between biotopes.

	Urban	Park	Hospital	Rural	Pig shelter
Urban		3.44	3.56	7.65	17.89
Park			4.67	10.37	15.36
Hospital				6.10	20.02
Rural					25.50
Pig shelter					

All distances in kilometers (km).

### The traps

Two traps were compared: (1) BGM trap (Biogents HmGb, Regensburg, Germany); (2) CDC light traps (Lucky Star Environmental Protection Technology Co., Ltd., Wuhan, China). The BGM trap is baited with the BG-Sweetscent (Biogents HmGb, Regensburg, Germany) and CO_2_. BG-Sweetscent is an artificial odor that mimics scents found on human skin. The primary constituents of BG-Sweetscent are lactic acid, ammonia, and fatty acids. A gas cylinder supplies CO_2_ at a flow rate of 0.5 kg/day (the recommended flow rate by the Biogents company). The CDC light trap attracts mosquitoes with the UV light. BGM traps were placed on the ground following manufacturers' instructions; the CDC trap was hung on trees or other supports (1.5 m above ground).

### Experimental design

Field trials started from May 21st, 2018 to November 31st, 2018. The sampling was conducted twice a month, with one on the 5st day of each month and the other on the 21st day of each month. Traps were set up 1 h before the sunset and removed 1 h after sunrise the next day; this was a sampling period. Each pair of traps (one BGM trap and one CDC lighting trap) were placed in five different biotopes in turn. At each biotope, two traps were separated 50 m from each other. In the next trapping period, the two traps rotate to the counterpart’s place to reduce sampling point specific differences. After the end of the trapping period, mosquitoes were brought back to the laboratory for classification and identification. By referring to Lu^22^, we identified morphologically all collected mosquitoes in the laboratory after the end of each trapping period. Due to the morphological similarity between *Cx. pipiens pallens* and *Cx. quinquefasciatus*, our research did not distinguish the two species, and instead, we summarized morphologically similar mosquitoes into *Cx. pipiens* sensu lato (s.l.).

### Statistical analysis

The dataset includes 1300 sex-specific observations, and 917 among 1300 recorded no mosquito, which indicates a risk of zero-inflation influence existing in buildings. To eliminate the effect of zero-inflation, a zero-inflated generalized linear mixed model was used to analyze the effect of the BGM trap device and the CDC trap device on total counts of mosquitoes caught per trap, counts of mosquitoes caught by species per trap, counts of mosquitoes caught by sex per trap, and counts of mosquitoes caught per trap in different biotopes. The dependent variable of our model is count data, which might fit Poisson distribution. However, the overdispersion test indicates our model is over-dispersed (*p* value < 0.001). To counteract the count data's overdispersion, zero-inflated negative binomial regression models were built in our study. We treat the test timing and biotopes as random variables to account for the variations in temperature, humidity, and other confounding effects during the study periods. All analyses and model examinations were conducted in R 3.6.3, with functions from the packages glmmTMB, tidyverse, emmeans, multcomp, ggplot2, DHARMa^23–29^. Sex ratio difference of trapped mosquitoes between CDC light traps and BGM traps were calculated by *X*^2^-test.

## Results

**Figure 2:**
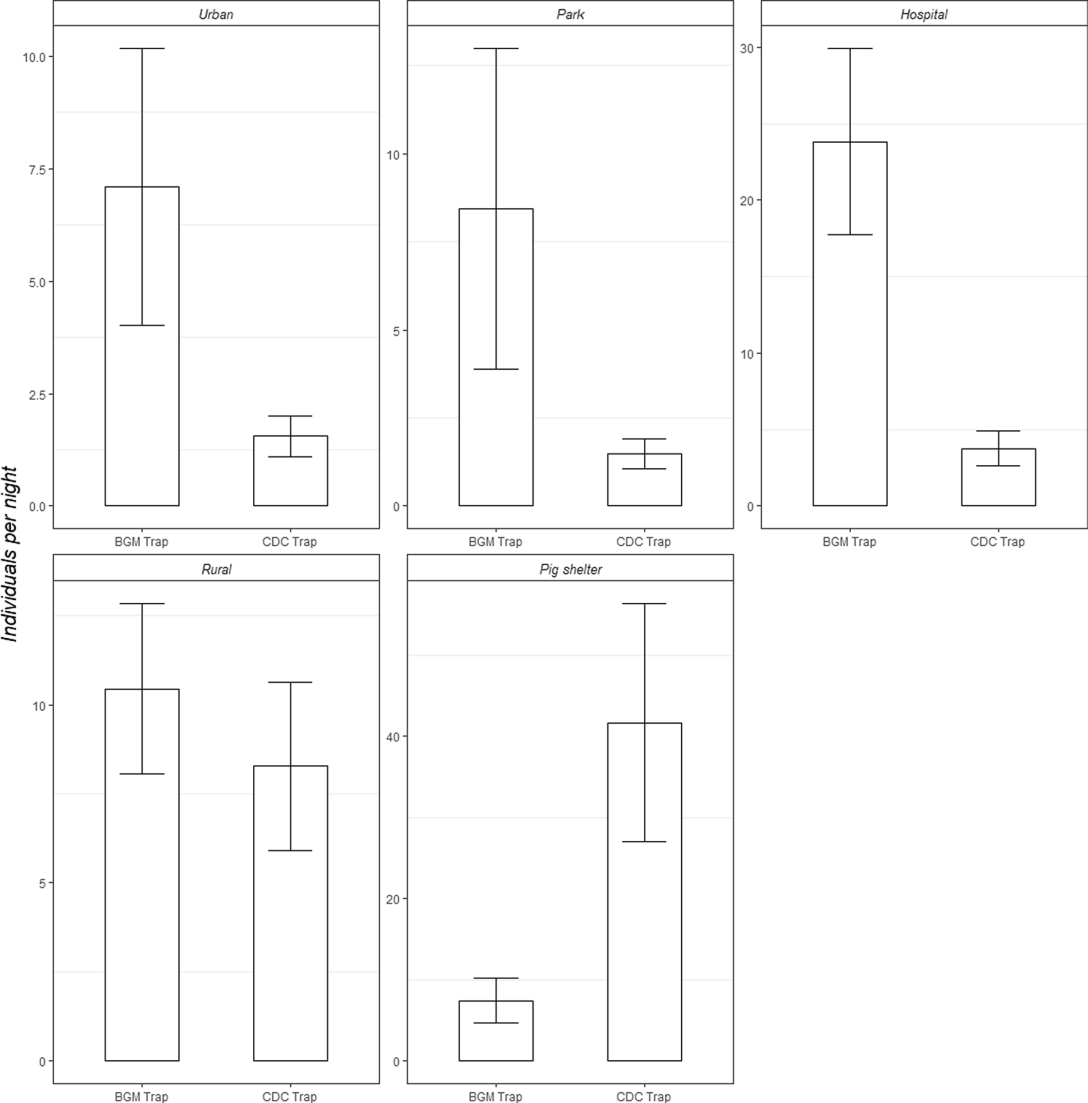
Average number of mosquitoes caught per sampling period by two types of trapping devices among five different biotopes.

A total of 13 sampling periods were completed across the study, with 65 trap comparisons evenly conducted in 5 different biotopes. We, with two different-type traps, collected 7406 mosquitoes in total during the study process, including 4636 *Cx. pipiens* s.l., 1183 *Cx. tritaeniorhynchus*, 1511 *Ae. albopictus*, 26 *An. sinensis*, and 50 *Ar. subalbatus* (Table 2). Both traps caught all five species by the end of this study, but the proportion of different mosquito species caught varied by traps (Table 2). The overall female-to-male sex ratios of individuals caught in the BGM trap, and CDC light trap, were both 4:1. Only for the *Cx. pipiens* s.l., the female-to-male sex ratio of the BGM trap was significantly higher than the CDC light trap (Table 2). During each trapping period, the BGM trap caught significantly more mosquito individuals than the CDC trap, in an urban residential area, park, and hospital (Table 3, Fig. 2). In contrast to comparisons in the other four biotopes, the CDC trap has a significantly better performance in the pig shelter (Table 3, Fig. 2).

**Table 2:** Number and percentage of mosquitoes caught by species and by sex with two types of trapping devices.

Species	BGM trap		CDC light trap		Sex ratio difference
	Count	Proportion (%)	Count	Proportion (%)	
***Cx. pipiens s.l***					
Female	1749	47.05%	1903	52.28%	X^2^ = 69.263
Male	325	8.74%	659	16.73%	*p* < 2.2e−16
***Cx. tritaeniorhynchus***					
Female	157	4.22%	906	24.89%	X^2^ = 3.794
Male	10	0.27%	110	3.05%	*p* = 0.0514
***Ae. albopictus***					
Female	993	26.72%	57	1.57%	X^2^ = 1.1458
Male	442	11.89%	19	0.55%	*p* = 0.2844
***An. sinensis***					
Female	10	0.27%	15	0.38%	NA
Male	0	0.00%	1	0.03%	
***Ar. subalbatus***					
Female	31	0.83%	19	0.52%	NA
Male	0	0.00%	0	0.00%	
**Total**					
Female	2940	79.10	2900	79.64	X^2^ = 0.33578
Male	777	20.90	789	20.36	*p* = 0.5623
Total	3717	100%	3689	100%	

Significance level in the two-tailed test sets as 0.05.

**Table 3:** Statistical difference of numbers of mosquitoes caught per sampling period by locations between two types of trapping devices.

Species	Method	Estimate	S.E.	t-value	*p* value
Urban	BG trap versus CDC trap	1.1183	0.3981	− 2.809	0.00497
Park	BG trap versus CDC trap	0.8828	0.3554	− 2.484	0.01300
Hospital	BG trap versus CDC trap	1.7706	0.5073	− 3.490	0.00048
Rural	BG trap versus CDC trap	0.2186	0.2629	− 0.831	0.40600
Pig shelter	BG trap versus CDC trap	− 1.9180	0.2866	6.692	2.2E−11

*S.E*. standard error. Significance level in the two-tailed test sets as 0.05.

**Figure 3:**
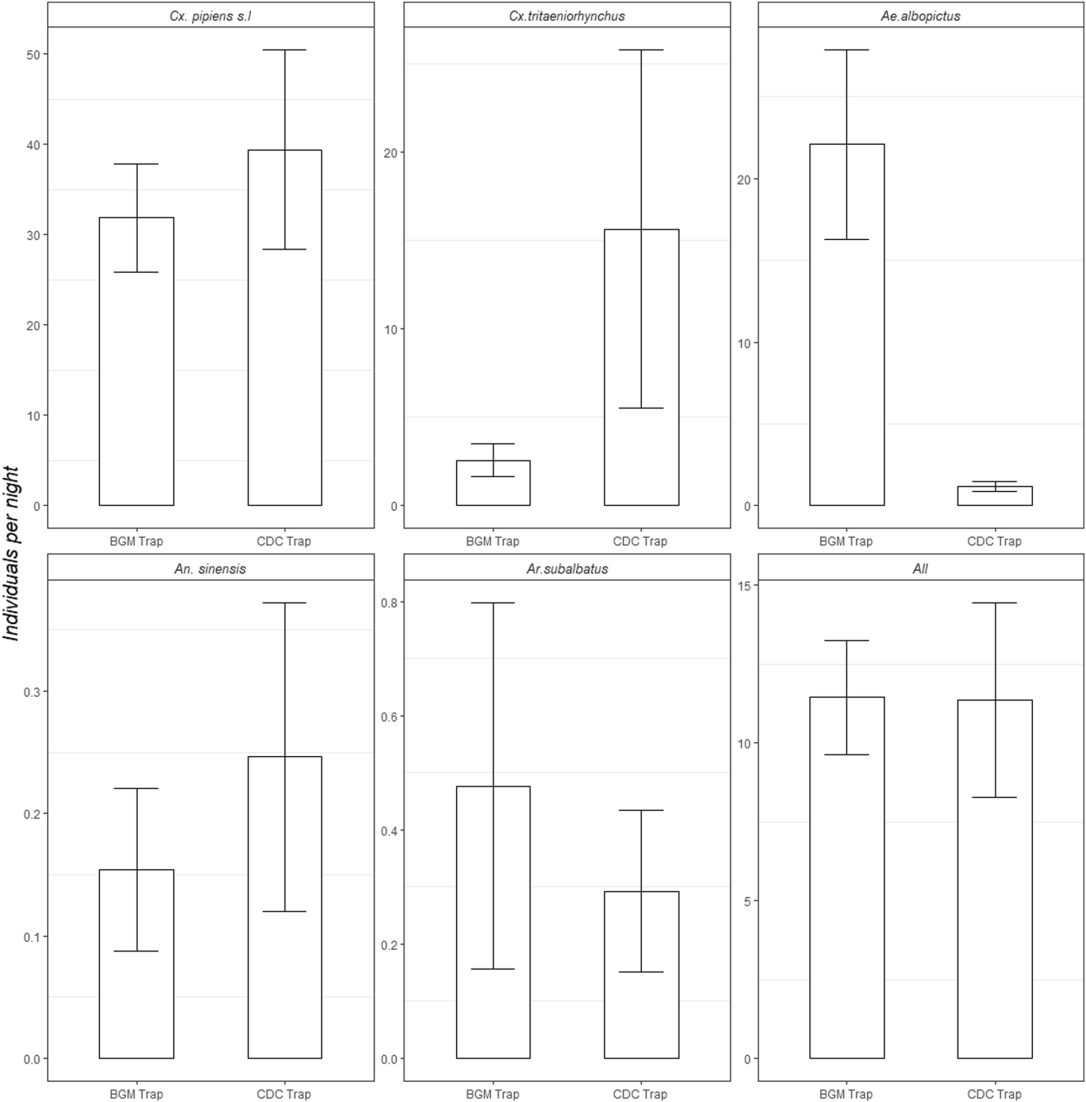
Average number of mosquitoes caught per sampling period per specie or all species by two types of trapping devices.

The performance of the BGM trap and CDC trap in catching mosquitoes varies by mosquito species (Table 4a,b, Fig. 3). In terms of capturing *Cx. pipiens* s.l., *An. Sinensis*, *Ar. subalbatus*, no significant difference between the BGM trap and the CDC trap was observed. As for *Ae. albopictus*, the BGM trap caught significantly more individuals than the CDC trap per trapping period (*p* < 0.001). By contrast, for *Cx. tritaeniorhynchus*, the CDC trap caught significantly more individuals each time (*p* = 0.0188). Due to the fact that adult male mosquitos do not bite humans, mosquito-borne disease transmission should be mainly attributed to adult female mosquitos. Therefore, our study separated the female and analyzed BGM and CDC traps' effect on trapping female mosquitoes specifically. Results show that the BGM trap's overall performance across all biotopes is better in trapping *Ae. albopictus* than the CDC trap (Table 4b).

**Table 4:** Statistical differences of (a) numbers of mosquitoes per sampling period, (b) numbers of female mosquitoes per sampling period by species between two types of trapping devices.

Species	Method	Estimate	S.E.	t-value	*p *value
**(a)**					
*Cx. pipiens* s.l	BG trap versus CDC trap	0.07515	0.25658	0.293	1
*Cx. tritaeniorhynchus*	BG trap versus CDC trap	− 1.45339	0.45891	− 3.167	0.0188
*Ae. albopictus*	BG trap versus CDC trap	2.16439	0.35104	6.166	< 0.0010
*An. sinensis*	BG trap versus CDC trap	− 0.14678	0.57030	− 0.257	1
*Ar. subalbatus*	BG trap versus CDC trap	0.44874	0.45575	0.985	1
**(b)**					
*Cx. pipiens* s.l	BG trap versus CDC trap	0.56394	0.28113	2.006	0.679357
*Cx. tritaeniorhynchus*	BG trap versus CDC trap	− 1.20605	0.47850	− 2.520	0.287196
*Ae. albopictus*	BG trap versus CDC trap	2.15010	0.39352	5.363	2.69E–06
*An. sinensis*	BG trap versus CDC trap	0.09139	1.05571	0.087	1
*Ar. subalbatus*	BG trap versus CDC trap	0.90617	0.93322	0.971	1

*S.E.* standard error. Significance level in the two-tailed test sets as 0.05.

**Figure 4:**
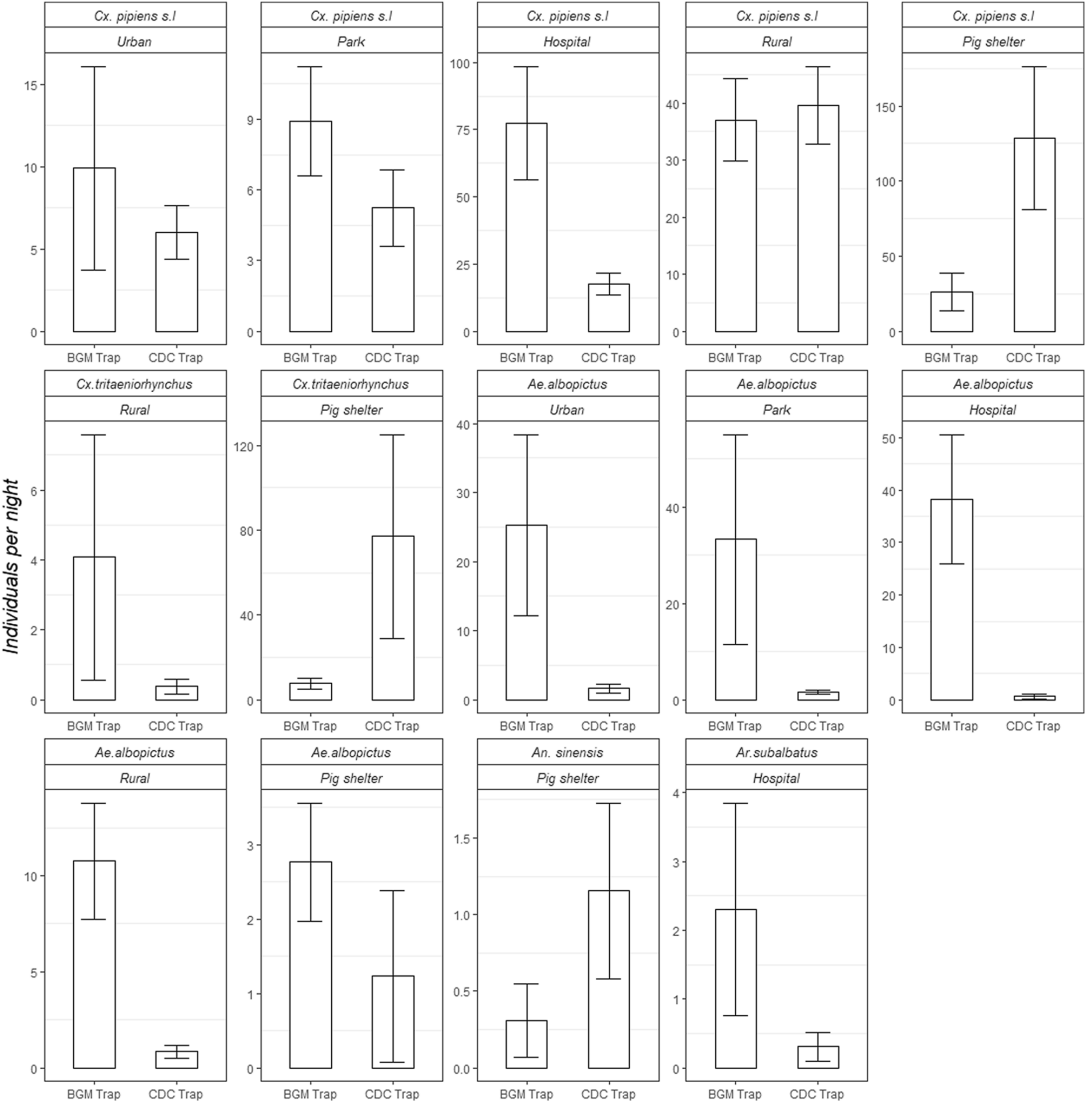
Average number of mosquitoes caught per sampling period per species in different biotopes by two types of trapping devices. Only comparisons including at least one value no less than 1.0 were shown.

**Figure 5:**
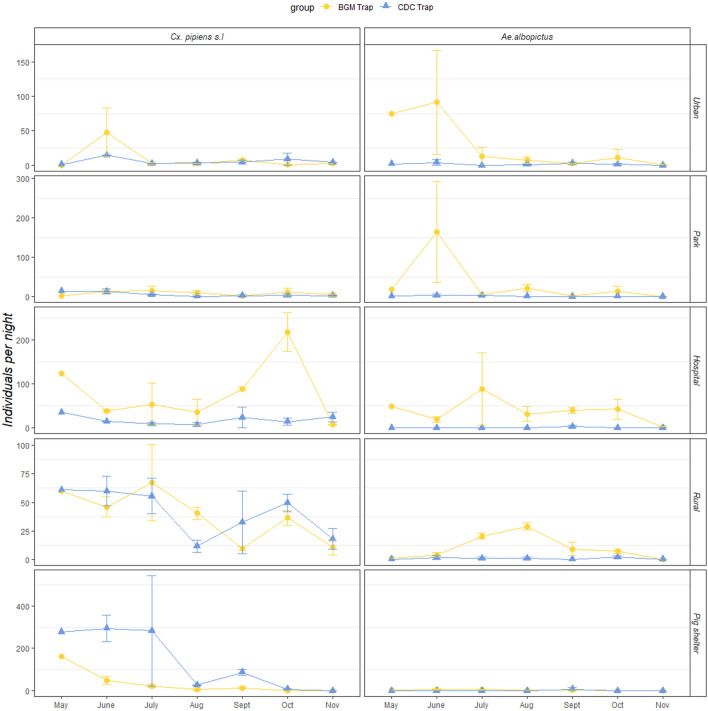
Population dynamics of *Cx. pipiens* s.l. and *Ae. albopictus* trapped by two types of trapping devices in five different biotopes. Points are mean values of numbers of individuals caught in specific month with 95% confidence intervals.

Also, even for the same species, the CDC trap and the BGM trap's performance can vary by biotopes (Fig. 4, Table 5a,b). Of note, for capturing *Ae. albopictus* (both sexes and female only), the BGM trap had a significantly better performance in all biotopes except the pig shelter. The population changes of *Cx. pipiens* s.l. and *Ae. albopictus* over time was shown in Fig. 5. For *Cx. pipiens* s.l., the number of captures by BGM traps does not significantly differ that by CDC traps, while more mosquitoes distribute in the hospital, the rural residential area, and the Pig shelter (Z = 5.258, 3.620, 2.484; *p* = 1.46e−07, 0.0003, 0.013, Table 6). Also, two traps depicted similar trends of population changes of *Cx. pipiens* s.l. (Fig. 5). For *Ae. albopictus,* the capture number of BGM traps is significantly more than the number of CDC traps (Z = −4.587; *p* = 4.50e−06; Table 6). Meanwhile, *Ae. albopictus* were less likely to be trapped in the pig shelter than the other four places (Z = −1.7575; *p* = 0.00128; Table 6, Fig. 5). The population change of *Ae. albopictus* is more conspicuous in BGM traps than in CDC traps (Fig. 5).

**Table 5:** Statistical differences of (a) numbers of mosquitoes per sampling period, (b) numbers of female mosquitoes per sampling period by species by different locations between two types of trapping devices.

Method	Specie	Biotope	Estimate	S.E.	t-value	*p *value
**(a)**						
BG trap VS. CDC trap	*Cx. pipiens* s.l	Hospital	1.4660	0.3797	3.861	0.00388
		Pig shelter	− 1.8230	0.3852	− 4.733	2.21E–06
	*Cx. tritaeniorhynchus*	Pig shelter	− 1.8342	0.5379	− 3.410	0.02064
	*Ae. albopictus*	Urban	2.5596	0.4784	5.351	5.01E−06
		Park	2.5803	0.4640	5.561	1.89E−06
		Rural	3.0048	0.5239	5.735	8.71E−07
**(b)**						
BG trap VS CDC trap	*Cx. pipiens* s.l	Hospital	1.7627	0.4392	4.013	0.00260
		Pig shelter	− 1.6286	0.4484	− 3.632	0.00811
	*Cx. tritaeniorhynchus*	Hospital	− 2.5851	0.9627	− 2.685	0.06451
		Rural	2.7734	0.6556	4.230	0.00076
		Pig shelter	− 1.7856	0.3411	− 5.235	2.15E−05
	*Ae. albopictus*	Urban	2.3234	0.6871	3.382	0.0300
		Park	3.1234	0.5523	5.655	5.32E−06
		Hospital	3.2534	0.8947	3.636	0.01399
		Rural	3.1268	0.6522	4.794	0.00020

*S.E.* standard error. Significance level in the two-tailed test sets as 0.05. Only significant differences were shown.

**Table 6:** Results of zero-inflated negative binomial regression analysis in the field study (for *Cx. pipiens* s.l. and *Ae. albopictus*).

Species	Coefficients	Estimate	S.E.	Z-value	*p* value
***Cx. pipiens s.l***					
	Intercept	2.0758	0.3466	5.989	2.12e−09
	CDC light trap	− 0.3412	0.4277	− 0.798	0.425070
	Habitat (Park)	0.1614	0.4328	0.373	0.70917
	Habitat (Hospital)	2.2344	0.4250	5.258	1.46e−07
	Habitat (Rural)	1.5171	0.4191	3.620	0.000295
	Habitat (Pig Shelter)	1.2171	0.4538	2.484	0.013006
***Ae. albopictus***					
	Intercept	2.5870	0.4678	5.530	3.21e−08
	CDC light trap	− 2.5802	0.5625	− 4.587	4.50e−06
	Habitat (Park)	0.1464	0.4963	0.295	0.76809
	Habitat (Hospital)	0.8037	0.5087	1.580	0.11411
	Habitat (Rural)	− 0.2112	0.5380	− 0.393	0.69465
	Habitat (Pig Shelter)	− 1.7575	0.5458	− 3.220	0.00128

## Discussion

Field evaluations of BGS traps have been conducted in studies all over the world, and most results show that BGS traps with CO_2_ baits and attractants are efficient in capturing multiple mosquito species including *Ae. Aegypti, An. Darlingi*, *Anopheles atroparvus, Aedes caspius,* etc^30–34^.

Researchers in mainland China also conducted researches with BGS traps, however, the trap’s performances are not consistent across studies. Liu et al.^35^ placed 20 BGS traps in Jinghong for a total of 240 h and only captured 26 mosquitoes. Chen et al.^36^ compared four adult mosquito monitoring methods in Yongcheng city, Henan province, and found BGS traps were inferior than CDC light trap in terms of *Cx. tritaeniorhynchus, An. sinensis, Ae. albopictus and Ar. Subalbatus*. In contrast, Li et al.^37^ found that the BGS is more effective than the CDC light trap in sampling adult *Ae. albopictus* (Z = −25.13, *p* < 0.001).

Since the BGM trap has the same mechanism as the BGS trap, the performance of the BGM trap in China needs to be studied. To now, only one research in China conducted a field evaluation of BGM traps and human-baited double net traps in trapping *Ae. Albopictus*, and the results show BGM traps are more efficient than the human-baited double net traps (t = 2.786, *p* = 0.006)^38^.

In our study, the different results depending on biotope could be partially explained by the difference in the two traps' working mechanism. The BGM trap captures mosquitoes by emitting CO_2_ with human skin odor, while the CDC trap uses light to attract mosquitoes. Pig shelters are usually dark and full of smell, and hogs within the shelter will produce a massive amount of CO_2_ through metabolism. In this environment, the BGM trap's odor can be largely diluted, and the gas would conceal CO_2_ emission from the trap within the pig shelter. In contrast, the CDC trap's light can easily catch mosquitoes’ attention in the dark environment. Thus, it seems clear that CDC light trap should be used to collect mosquitoes in pig shelters, while the BGM trap can be used in other biotopes where the trap’s odor and CO_2_ emission will not be interrupted.

Consistent with a comparative evaluation in Guangdong Province, China that compares the BGS trap to other-brand traps, our study found that the BGM trap caught significantly more *Ae. albopictus* than the CDC trap^37^. Our further analysis found that in all biotopes except pig shelter, BGM traps all outperformed CDC traps in terms of amount of *Ae. albopictus* trapped. The exception could still be explained by pig shelter's unique characteristics, which impeded the BGM trap mechanism. Moreover, our finding that the number of individuals caught differs by sex is similar to other field evaluation’ findings in Guangdong Province^37^. This phenomenon could be explained by the biological difference between female and male mosquitoes that the human-skin odor is more attractive to females than males. The male mosquitoes usually stay in the wild and eat nectar, rather than suck human blood in areas where humans live^39^.

In our study, the overall performance of the BGM trap is not significantly different from that of the CDC trap in trapping *Cx. pipiens* s.l*.* (*p* = 0.5623) (Table 3). This result is inconsistent with a Germany study that the BG trap is superior to any other traps, including CDC light traps, in capturing *Cx. pipiens* s.l., but consistent with a Spanish study and a Dutch study^30,40,41^. The performance of traps varies across biotopes. We found the same situation that the CDC trap outperformed in the pig shelter environment. However, the BGM traps inversely outperformed in the hospital environment. The former phenomenon can still be attributed to the mechanism difference between two traps, while the latter mechanism remains unclear. We researchers speculated that numerous lighting devices in the hospital would keep turning on even at midnight, which indirectly helps BGM traps intrigue mosquitoes.

To represent the population dynamics of the most common mosquito species in Yiwu city (*Cx. pipiens* s.l. and *Ae. albopictus*), our study used both BGM traps and CDC traps to monitor the populations’ fluctuation of these two mosquito species in five biotopes (Fig. 5, Table 6). Our study shows that BGM traps were more sensitive to the population change of *Ae. albopictus*, except in the pig shelter; while BGM traps can depict better, or at least equivalent, fluctuations of *Cx. pipiens* s.l. numbers than CDC traps do, except in the pig shelter. All these findings suggest the BGM trap is suitable for mosquito surveillance in China.

The price of BG-Mosquitaire CO_2_ Bundle, consisting of a BGM trap, a BG-Sweetscent (lasting for 2 months), and a BG-Booster CO_2,_ is $ 279.00^42^. Since the Bundle does not include a CO_2_ gas cylinder which costs about $70.00 for 5 lb., the total price of operating a BGM trap is $350.00. By contrast, the price of a domestic CDC light trap is only $30.00. Due to the high cost and relatively late development, the BGM trap has not been ever used in Chinese mosquito surveillance until now. However, our study has shown that the BGM trap is more effective than the CDC light trap in collecting *Ae. albopictus*, the recognized vector of Dengue virus, therefore should can be used in the dengue surveillance. Furthermore, for the other four common mosquito species in China, no significant difference in effectiveness between the BGM trap and CDC trap was detected in four out of the five typical biotopes (except in Pig shelter). Given the limited involvement of pig shelters, compared to urban/rural residential areas, parks, and hospitals, we can say the BGM trap is more suitable than the CDC light trap to be used in daily mosquito surveillance.

Our study has several limitations: (1) Our study did not set replicates for the field evaluation in five biotopes over the study periods due to limited human resources. Thus, the representativeness of mosquito distribution in five biotopes and the and external validity of the effectiveness of mosquito trapping devices in each biotope was decreased. (2) Our study aims to compare the effectiveness of mosquito trapping methods commonly used in Chinese mosquito surveillance to BGM traps. In other words, we simulate the operation by not adding CO_2_ bait to CDC light traps. However, in this study, to maximize BGM traps’ function, we added both BG-Sweetescent bait and CO_2_ bait to BGM traps. The difference in equipped bait could potentially influence the results by intrinsically larger attractant effect of BGM traps. Thus, future study should also include a group of CDC light traps with CO_2_ bait as a comparison.

## Conclusion

We recommend that researchers and institutions with enough financial capacity to take advantage of the BGM trap to monitor mosquito and further control local arbovirus transmission.
